# P-343. Impacts of an Implementation Science Initiative to Support Comprehensive Care Coordination for People Living With HIV in Two Community HIV Clinics

**DOI:** 10.1093/ofid/ofaf695.561

**Published:** 2026-01-11

**Authors:** Ellen Eaton, Claudia Martorell, Tanya Schreibman, Kelly E Pillinger, Leah Molloy, Laura Simone, Chris Napolitan, Chelsie Chadha, Melissa Rodriguez, Jeffrey D Carter, Bonnie Douglas

**Affiliations:** University of Alabama, Birmingham, Birmingham, Alabama; The Research Institute; 3CAN Community Health, Sarasota, Florida; PRIME Education, New York, NY; PRIME Education, New York, NY; PRIME Education, LLC, Fort Lauderdale, Florida; PRIME Education, New York, NY; PRIME Education, New York, NY; PRIME Education, New York, NY; PRIME Education, LLC, Fort Lauderdale, Florida; PRIME Education, LLC, Fort Lauderdale, Florida

## Abstract

**Background:**

This project aimed to support comprehensive care coordination using tenants of the patient-centered medical home model in 2 community HIV clinics in MA and FL. Interventions included 1) a coordinated care toolkit (CCT) for clinic staff and 2) a digital health program (app) to support people living with HIV between visits.

**Table 1.**
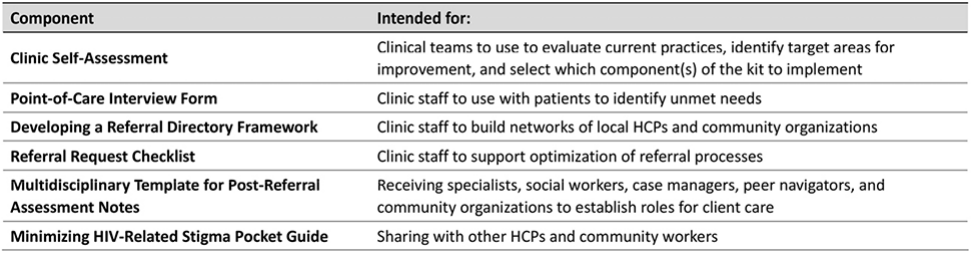
Components of the Coordinated Care Toolkit.

**Table 2.**
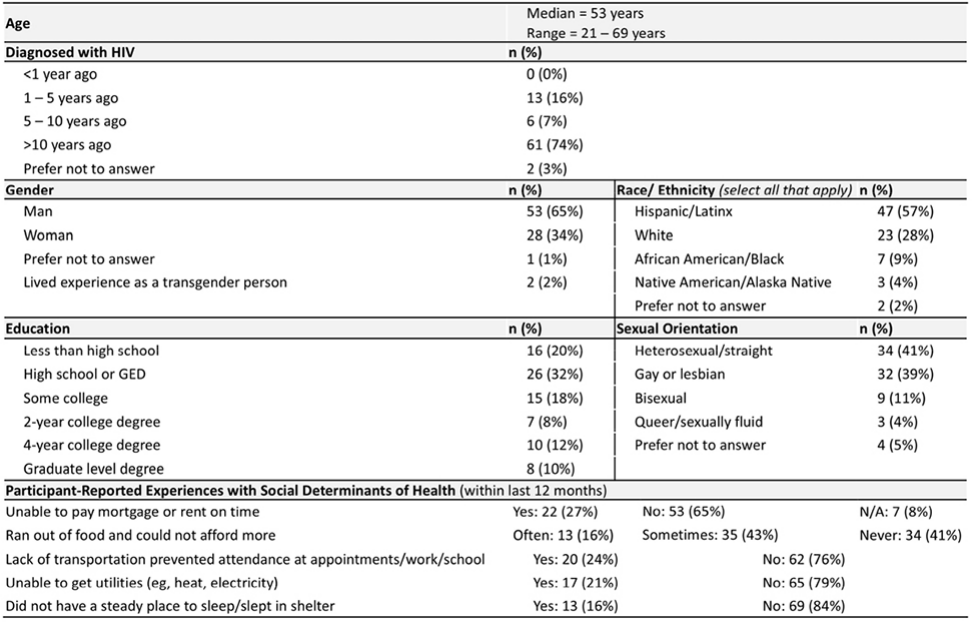
Baseline Characteristics of Participants in the Digital Health Program (app) (N = 82).

**Methods:**

Clinics implemented a CCT developed to address challenges identified by their staff, regional specialists, and local community organizations (Table 1). Concurrently, clinics enrolled adults living with HIV in a 12-week app through Reciprocity Health®, including biweekly education on HIV treatment options, navigating care, and overcoming stigma along with patient-reported outcome (PRO) surveys. EMR audits evaluated patient health measures before and after implementation of the CCT and app.

**Table 3.**
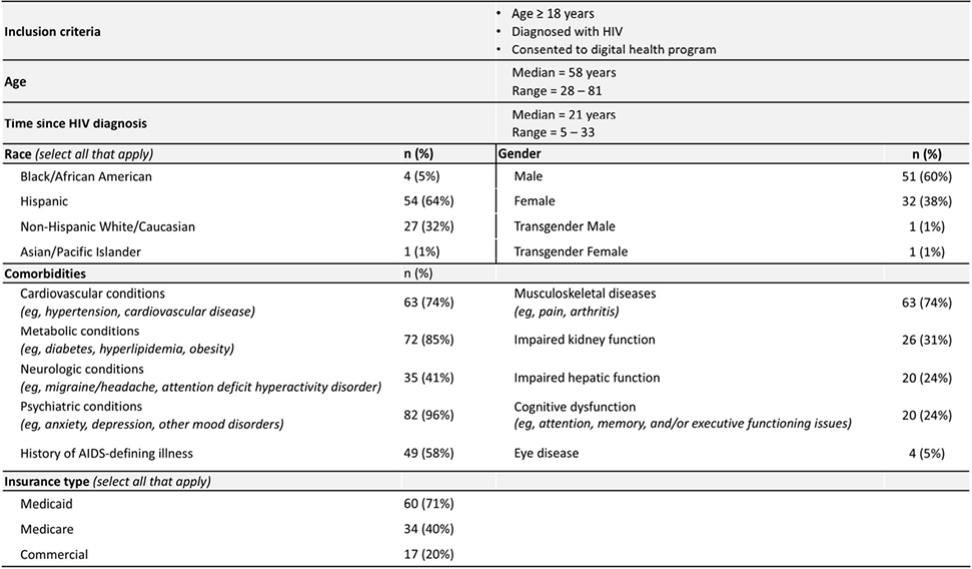
Baseline Characteristics of Patients Included in the EMR Audit (N = 85).

**Figure 1.**
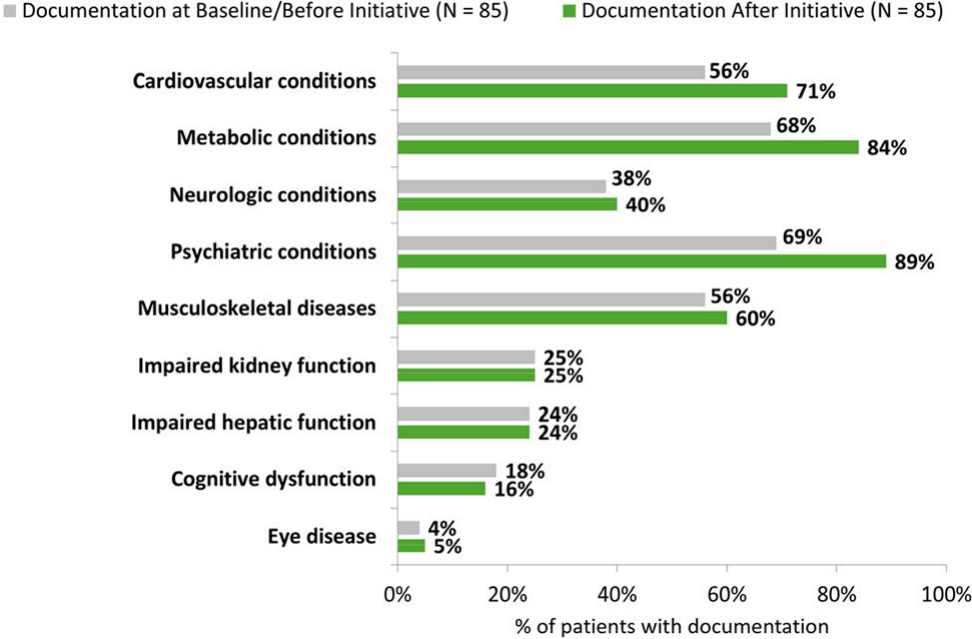
Documentation of Comorbidity Management Before and After Implementation of the Coordinated Care Toolkit and Digital Health Program (app).

**Results:**

As of April 2025, 123 participants consented to participate in the app across clinics: 82 were actively engaged and 47 had completed the program (Table 2). From week 0 to 12, there was a numerical decrease in participants who reported being very/extremely worried about their health (37% to 21%, p = .065) or having physical pain disrupt their routine (27% to 14%, p = .101). At completion, 77% said the program increased their confidence in meeting health goals, 74% reported increased knowledge of treatment options, and 70% felt more confident sharing preferences/goals/barriers during visits. EMR audits were available for 85 patients who consented to the app in one clinic as of April 2025 (Table 3). After the initiative, more patients had documentation of certain comorbidity management (Fig 1), more were adherent to ART (72% to 94%, p < .001), and fewer reported side effects (47% to 23%, p < .001) than at baseline. Over the initiative, 31 patients switched ART, with 21 regimen simplifications. Four patients had detectable viral loads after the initiative versus 8 at baseline. Clinics documented asking patients about concerns, fears, and barriers to treatment goals more often during the initiative than before (76% to 98%, p < .001).

**Conclusion:**

The digital health app enhanced patient quality of life, confidence, and knowledge. EMR audits from one of two clinics suggest these combined interventions support ART adherence, comorbidity management, and shared decision-making.

**Disclosures:**

Ellen Eaton, MD, MPH, Gilead: Honoraria Claudia Martorell, MD, MPH, FACP, AbbVIe: Grant/Research Support|AbbVIe: Speaker Bureau|ATEA: Grant/Research Support|Gilead: Grant/Research Support|Gilead: Speaker Bureau|Theratechnologies: Grant/Research Support|Theratechnologies: Speaker Bureau|ViiV: Grant/Research Support|ViiV: Speaker Bureau

